# Validation of the Gothenburg obstetric triage system

**DOI:** 10.1002/pmf2.70358

**Published:** 2026-07-07

**Authors:** Linnéa Lindroos, Erica Ginström Ernstad, Staffan Nilsson, Verena Sengpiel

**Affiliations:** ^1^ Department of Obstetrics and Gynaecology Region Västra Götaland Sahlgrenska University Hospital Gothenburg Sweden; ^2^ Department of Obstetrics and Gynaecology Institute of Clinical Sciences Sahlgrenska Academy University of Gothenburg Gothenburg Sweden; ^3^ Institute of Biomedicine University of Gothenburg Gothenburg Sweden

**Keywords:** acuity, emergency care, obstetric, triage, validity

## Abstract

**Introduction:**

Obstetric emergency triage is a relatively new form of emergency triage and not yet fully implemented within obstetric emergency care. In addition to assessing both the pregnant patient and fetus, obstetric triage systems require adaptation to the physiological changes of pregnancy. Without adapted triage systems, obstetric patients are known to receive unequal care compared to non‐obstetric emergency patients, resulting in avoidable adverse outcomes. This study aims to validate the Gothenburg obstetric triage system, a five‐level obstetric emergency system based on symptom presentations and vital sign parameters.

**Materials and methods:**

Consecutive medical charts from the obstetric emergency department at Sahlgrenska University Hospital between January 1–17 and June 1–17, 2021 were reviewed. Triage levels were dichotomized into urgent (red/orange) and nonurgent (yellow/green/blue) and assessed in relation to 31 previously established acuity outcome measures reflecting acuity in the moment of triage. Sensitivity and specificity as well as over‐ and undertriage were assessed for Gothenburg obstetric triage system (GOTS) in general and individual chief complaints. For undertriaged patients, deepened chart reviews were performed.

**Results:**

Of 1280 patient visits, the appropriate triage level had been assigned in 96% of the patient visits; 2.3% (29/1280) were undertriaged. Sensitivity and specificity were 0.62 (confidence interval [CI], 0.50–0.73) and 0.98 (CI, 0.97–0.99), respectively. Subjective symptom descriptions such as pain and dyspnea were prone to under‐ and overtriage.

**Conclusion:**

We suggest a two‐phase validation process with standardized acuity outcome measures and deepened chart reviews to assess validity of obstetric triage systems in a given obstetric context. We found that GOTS is a valid triage system in the studied context but also identified areas of further improvement.

## 1 INTRODUCTION

Obstetric emergency triage (OET) is a relatively new branch of emergency triage, with approximately 10 different obstetric triage systems (OTSs) developed worldwide since 2010 [1, 2]. Like non‐OET, OET constitutes a working method in the emergency setting, enabled by OTSs. It aims to standardize initial assessment of the patient and hereby identify and prioritize patients with urgent and time‐dependent ailments, ensuring timely interventions and effective resource utilization [3, 4, 5]. OET is more specialized than general emergency triage. It requires assessment of the pregnant patient and fetus as well as labor status; moreover, OTSs must be adapted to the obstetric patient's physiological changes [6, 7, 8]. Unlike non‐OET, it is not yet an implemented and manifest part of obstetric emergency care. Instead, patients are assessed according to time of arrival or by subjective standards based on the caregivers’ clinical experience.

Mapping decision‐making processes in the emergency department (ED), Calder et al. found triage to be one of the most error prone activities in the ED [9]. In the general ED setting, obstetric patients are known to receive unequal care compared to the non‐obstetric patients, repeatedly leading to avoidable adverse outcome [10, 11, 12]. This inequality is caused by both insufficient knowledge of the obstetric patient group including physiological changes during pregnancy and the postpartum period as well as inappropriate triage systems. In the obstetric setting, patients seeking obstetric emergency care are often seen in labor departments and, hence, prioritized in comparison to patients in active labor. In 2017, the American College of Obstetricians and Gynecologists urged hospital‐based obstetric units and EDs to establish guidelines for triage of pregnant patients [13]. Since then, an acknowledgement of the necessity to assess and prioritize obstetric emergency patients has led to an increasing interest in OET, both in Sweden and other European countries.

Previous research has shown extensive gaps in establishing validity in both general triage systems and OTSs [1, 14, 15]. Validity is commonly assessed by sensitivity and specificity in relation to a “true positive outcome.” Lacking a definition of true acuity in a patient seeking emergency care, triage validity has rather been assessed by prediction of surrogate outcome measures. Good validity is thus claimed by presenting a correlation between triage level and, for example, length of stay in the ED, hospital admission, or resource utilization. These outcome measures are highly influenced by external factors unassociated with the patient's acuity at the moment of triage and have therefore repeatedly been questioned [15, 16, 17]. In addition, due to different definitions on outcome measures, results are often incomparable [15]. To our knowledge, only one previous OTS has undergone such an assessment presenting a sensitivity and specificity of 75% and 77%, respectively [2]. However, the study was based on the inapt outcome measures and included no further analysis or discussion of the results.

Several previous papers have suggested that a consensus‐focused, multi‐stage Delphi method could be used to define more adequate outcome measures, assessing the actual acuity and need for intervention at the triage moment [18, 19, 20, 21]. Two recent Swedish publications report outcome measures developed via a Delphi process, one of which is adapted to general emergency care [22] and the other to OET [23]. In the latter, a set of 31 actions (e.g., treatment or medication orders), performed or initiated at the ED by the obstetrician and/or midwife, are graded into three levels: *directly lifesaving* such as cardiopulmonary resuscitation (CPR), immediate cesarean section, or thrombolysis; *potentially lifesaving* such as antibiotic infusion, magnesium infusion, or operation within 30 min; and *not directly lifesaving* such as admission with surveillance of blood pressure or echocardiogram. The outcome measures thus represent the acuity of a patient's condition, evaluated in direct proximity to triage, by the initial caregiver's assessment in the ED setting [23].

The Gothenburg obstetric triage system (GOTS) is a five‐level triage system based on 14 chief complaint algorithms (CCAs). It includes assessment of vital sign parameters and symptoms and additionally provides direction on initial management. Previous studies present the development of GOTS and have shown good interrater reliability [24, 25]. This study aims to establish GOTS validity according to a reference standard of acuity outcome measures [23] and further to assess reasons for mis‐triage.

## 2 METHOD

### 2.1 Setting

**FIGURE 1:**
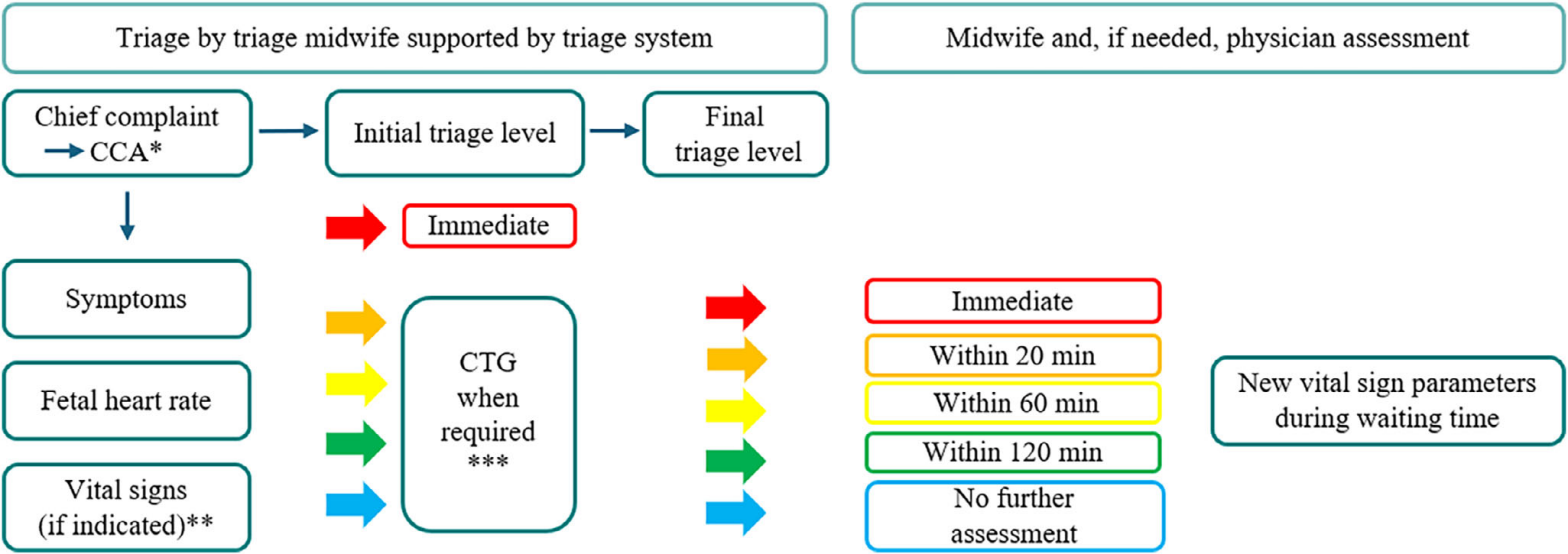
Triage process in obstetric emergency triage, visualizing the initial and final triage levels. * Chief complaint algorithm. ** Vital sign parameters are mandatory when the patient is triaged as green or more urgent by symptoms. *** CTG is performed from gestation week 24+0 if none reassuring fetal heart rate by doppler or if symptoms indicate the need for fetal assessment by CTG. CTG, cardiotocography.

The study is a retrospective cohort study performed at a single, tertiary referral hospital, facilitating approximately 10,000 annual deliveries. The unit has an obstetric ED with about 14,000 annual unscheduled, obstetric emergency visits between gestational week 18+0 and 12 weeks postpartum and has worked with OET since 2017 when GOTS was implemented. Triage is performed by midwives, the triage process being divided into two phases with a first assessment of the pregnant patient and fetus (by auscultation of fetal heartbeat) and a final assessment taking cardiotocography (CTG) into account, depicted in Figure 1. Midwives in Sweden are educated according to the International Confederation of Midwives (ICM) criteria [26] and manage cases such as prelabor rupture of membranes, reduced fetal movement (selected cases), and early onset of labor at the obstetric ED without obstetrician involvement (approximately 40%). If the final triage level is affected by abnormal CTG, the patient is automatically triaged into one of the two highest levels of triage acuity, and an obstetrician is immediately involved. Obstetric patients with circulatory and/or respiratory failure or neurological symptoms indicating stroke are directed to the general ED.

### 2.2 Data collection

Medical charts of 1280 consecutive visits at the obstetric ED, in two separate time periods, January 1–17, 2021 and June 1–17, 2021, were reviewed. These time periods were chosen to cover seasonal changes in infectious diseases including Covid‐19. Data on timelines during visits such as time of arrival, time of midwife/obstetrician assessment, and time of admission as well as triage level were collected from electronic medical journal systems. From the chart review, information on patient characteristics (Table 1), triage level as well as prespecified acuity outcome measures (previously described) have been assessed. Data collection was performed by the first author. The second author then verified 10% of the data by a second review of every 10th patient visit in the cohort. With only 0.3% of the data items not being in agreement (21/6784), the dataset was deemed reliable. The mismatching data points were corrected by consensus discussion between all authors.

**TABLE 1:** Patient characteristics, *n* = 1280 visits to the obstetric emergency department.

	Number *(%)*	SD
Age at ED visit		
<20	7 *(0.5)*	
20–29	454 (*35.5)*	
30–39	752 *(58.7)*	
>40	67 *(5.2)*	
Mean	31.7	4.8
BMI		
<18.5	33 *(2.6)*	
18.5–24.9	607 *(47.4)*	
25–29.9	386 *(30.2)*	
≥ 30	214 *(16.7)*	
*≥40*	*15 (1.2)*	
Mean	25.1	1.4
*Missing*	40 *(3.1)*	
Smoking at first visit to antenatal care		
Yes	64 *(5.0)*	
No	1168 *(91.3)*	
*Missing*	48 *(3.8)*	
Work Employment at first visit to antenatal care		
Employed	892 *(69.7)*	
Unemployed or parental leave	355 *(27.7)*	
*Missing*	33 *(2.6)*	
Parity		
0	582 *(45.5)*	
1	470 *(36.7)*	
≥2	228 *(17.8)*	
Gestational length at ED visit		
<21+6	50 *(3.9)*	
22+0–27+6	126 *(9.8)*	
28+0–36+6	447 *(34.9)*	
≥37+0	459 *(35.9)*	
Postpartum	198 *(15.5)*	

Visits with missing triage levels were retrospectively triaged by a senior triage midwife blinded to clinical assessment and outcome after triage to enable inclusion in the study.

### 2.3 Statistics

Triage levels were dichotomized into urgent (red/orange) and nonurgent (yellow/green/blue). Categorical data are presented as numbers and percentages, continuous data as median and range or mean and standard deviation. Sensitivity and specificity were calculated with 95% confidence intervals (CIs).

Triage level was defined appropriate when a patient triaged red/orange (urgent level) had an acuity outcome or when a patient triaged yellow/green/blue (nonurgent level) did not have an acuity outcome. Undertriage was defined as a patient with acuity outcome triaged into a nonurgent level, as described in the introduction section [23]. Overtriage was defined as a patient without acuity outcome triaged into the urgent level. For cases identified as under‐ or overtriaged, deepened chart reviews were performed by L.L., E.G.E., and V.S. as re‐triage due to abnormal CTG might not be registered in the administrative triage system. In these cases, the clinical management of the patient was assessed as well to determine whether the clinical management was in concordance with the administrative triage level retrieved in data collection or not. If clinical management was deemed to correspond with a different triage level, for example, immediate summon of an obstetrician for acute assessment, the triage level was updated in accordance with clinical management.

Overall sensitivity and specificity were assessed. Moreover, sensitivity and specificity for all CCAs with more than 50 patient visits were assessed separately to identify potential areas in need of improvement.

## 3 RESULTS

Of 1280 patient visits, 98.4% had a registered triage level in the administrative system. Missing triage levels for the remaining 21 patients were mainly found in the nonurgent blue category (blue = 14; green = 3; yellow = 3; orange = 1) according to the retrospective blinded triage by a senior triage midwife. Baseline characteristics for patients and visits are presented in Tables 1 and 2.

**TABLE 2:** Emergency department (ED) visit characteristics, *n* = 1280 visits.

	Number *(%)*
Presentation to the ED	
Seeking on own initiative	
*Self‐ambulatory*	1130 *(88.3)*
*Ambulance*	15 *(1.2)*
Referral from other health institution	134 *(10.5)*
Other	1 *(0.07)*
Care provider	
Midwife only	531 *(41.5)*
Midwife and physician	749 *(58.5)*
Triage visit	1259 *(98.1)*
Complete vital sign parameter control^a^	
*Level 1—Red*	7/13 *(54)*
*Level 2—Orange*	25/46 *(54)*
*Level 3—Yellow*	95/236 *(40)*
*Level 4—Green*	226/485 *(47)*
*Level 5—Blue*	not applicable
Missing parameters in incomplete triage ^b^	
Blood pressure	7 *(1.6)*
Breathing frequency	420 *(98.4)*
Saturation	14 *(3.3)*
Pulse	11 *(0.9)*
Temperature	34 *(8)*
ED times	Median hh:mm, *(range)*
Time to triage	00:10 *(00:00–02:20)*
*Missing*	*36*
Time to midwife	00:16 *(00:00–03:07)*
*Missing*	*102*
Length of stay at the ED	02:25 *(00:02—13:22)*

^a^ Vital sign parameters are mandatory when the patient is triaged as green or more urgent by symptoms.

^b^ Adds up to >100% as several vital sign parameters may be missing at the same visit.

All patients are seen by a midwife after triage (Figure 1). After assessment, the midwife decides if the patient should be assessed by an obstetrician as well. Median time to assessment by the midwife after triage was 16 min for all patients. For patients triaged as urgent and nonurgent, the median time to assessment by the midwife was 8 and 16 min, respectively (Table 2).

**FIGURE 2:**
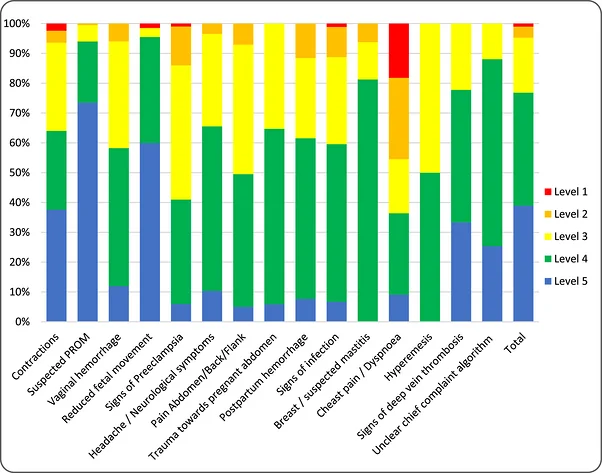
Distribution of triage levels within each chief complaint algorithm and in total (*n* = 1280 visits to the obstetric emergency department). Levels 1 and 2 are defined as urgent, Levels 3–5 are defined as nonurgent. PROM, prelabor rupture of membranes.

Of all patients, 59 (4.6%) patients were triaged as urgent, presented in Figure 2, also presenting the distribution of acuity levels within each CCA group.

The appropriate triage level was found in 96% (1229/1280), including the 21 retrospectively triaged patients. Thirty‐nine patients were initially found to be undertriaged; however, after chart review, it was evident that 10 of the 39 cases had been clinically managed as an urgent case (an obstetrician had been summoned immediately), eight of which were due to abnormal CTG. These 10 cases were deemed as correctly triaged even though the administrative triage level based on initial triage was incorrect. Including these data, GOTS had a sensitivity of 0.62 (CI, 0.50–0.73) and specificity of 0.98 (CI, 0.97–0.99), Table 3.

**TABLE 3:** Sensitivity and specificity for each chief complaint algorithm with more than 50 registered patient visits.

Chief complaint algorithm	Patient visits (*n*)	Clinically re‐triaged (*n*)	Sensitivity (CI 95%)	Specificity (CI 95%)
Reduced fetal movement	422	4	0.64 (0.35–0.87)	0.99 (0.99–1.0)
Suspected PROM	200	4	1.0 (0.48–1.0)	1.00 (0.98–1.0)
Contractions	125		0.43 (0.1–0.82)	0.96 (0.90–0.99)
Pain Abdomen/Back/Flank	100	1	0.36 (0.13–0.65)	0.97 (0.90–0.99)
Signs of preeclampsia	98		0.77 (0.46–0.95)	0.96 (0.90–0.99)
Signs of infection	89		0.67 (0.30–0.93)	0.95 (0.88–0.99)
Vaginal hemorrhage during pregnancy	67		0.5 (0.01–0.99)	0.95 (0.87–0.99)
Unclear chief complaint algorithm	67		^a^	1.0 (0.95–1.0)
Total	1168	9	**0.62** (0.49–0.73)	**0.98** (0.97–0.99)

Abbreviations: CI, confidence interval; PROM, prelabor rupture of membranes.

^a^ There were no patients in the unclear chief complaint algorithm with an urgent triage level.

Overtriage was mainly due to premature contractions with either unaltered cervical status or with previously administered steroids, hypertension with effective treatment administered at the ED and hence avoiding admission as well as patients with mastitis triaged to an urgent level due to high fever but able to return to home with antibiotics and antipyretics.

Of the 29 truly undertriaged patients, none were deemed to have been at immediate, obvious risk of adverse outcome. Diagnostic imaging or laboratory testing performed via the ED constituted the acuity outcome measure in 13 of the undertriaged patients. Ten patients were admitted with either steroids and/or atosiban, antibiotics, or antihypertensive treatment. All received treatment (pain relief, antibiotics, antihypertensive treatment) at the ED.

**FIGURE 3:**
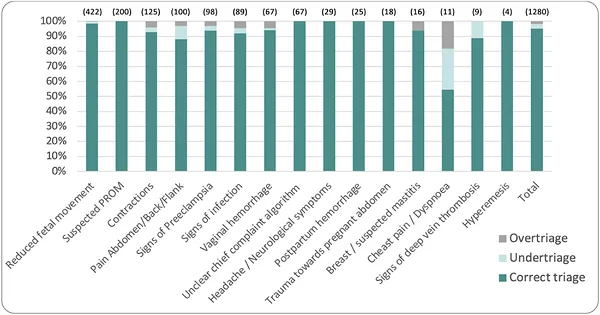
Percent of correct, over‐, and undertriage by each chief complaint algorithm and in total (*n* = 1280 visits to the obstetric emergency department). (*n*) = Patient visits within each specified chief complaint algorithm. Undertriage—patient triaged into one of the three lower triage levels despite an acuity outcome [23]. Overtriage—patient triaged into one of two highest triage levels despite not having an acuity outcome [23]. PROM, prelabor rupture of membranes.

To further assess whether a particular CCA was liable to mis‐triage, a separate assessment of under‐ and overtriage for each CCA, as well as a sensitivity and specificity assessment for each CCA assessing more than 50 patient visits, was performed (Figure 3). CCAs based on subjective symptom description such as pain, dyspnea, or contractions were prone to both under‐ and overtriage.

In 67 cases, the triage midwife did not find a CCA applicable to the patient's symptoms, for example, severe itching or pain in a perineal tear postpartum. In these cases, vital sign parameters in combination with clinical knowledge constituted the base for triage level.

## 4 DISCUSSION

Establishing a sensitivity of 62% and specificity of 98%, this study is, to our knowledge, the first validation of an OTS using a reference standard of acuity outcome measures [23]. When working with OET using GOTS, 96% of the patients received an appropriate triage level.

As there is no previous research on OET sensitivity and specificity with standardized outcome measures reflecting acuity at the moment of triage, the results cannot be compared to other triage systems and there are no data on how patients would fare without using obstetric triage at all. In a systematic review on non‐obstetric triage systems, both sensitivity and specificity were highly variable within each of the evaluated triage systems depending on which outcome measure had been used for evaluation. With GOTS having a specificity of 98%, patients triaged to the lower levels of acuity can safely wait without risk of adverse outcome [15]; however, further research is needed to understand cases of undertriage and increase sensitivity.

Triage systems aim at being precise; nonetheless, over‐ and undertriage will occur. For the individual patient, undertriage is the most serious mis‐triage, risking untimely interventions and prolonged waiting time. Overtriage on the other hand may reduce the sorting ability of the system with risk of overlooking the truly urgent patients. In this study, under‐ and overtriage was seen in 2.3% and 1.7% of patient visits, respectively. This is superior to previous studies on OTSs showing higher numbers of both under‐ and overtriage (Veit‐Rubin et al. 12% and 9.6%, respectively, and Moudi et al. 17.1% and 6.1%, respectively) [2, 27].

One contributor to mis‐triage was the difficulties of assessing the fetus's well‐being, a known aggravating factor in obstetric triage compared to general emergency triage [6, 12, 14]. As shown in Figure 1, OET is a two‐phased assessment. In this study, an initial triage level was found to be administratively registered after assessment of the pregnant patient but before CTG. Of the patients undertriaged according to initial triage level, 10/39 cases were clinically managed in accordance with a higher triage level, after fetal assessment. When fetal distress becomes evident, clinical management is prioritized compared to administrative changes in triage level. This clearly depicts the challenges in validating triage systems based on registered administrative data alone.

In accordance with previous findings, the triage process is often not fully documented due to missing registration of one or more vital sign parameters [25]. Whether this is equivalent to not taking the vital sign parameter into consideration is unknown. In this study, breathing frequency was almost never registered. There is very little research on normal vital sign parameters in the pregnant patient, and the role of each vital sign parameter on triage validity should be a focus area in future research.

The study confirms that CCAs based on subjective symptom description such as pain, dyspnea, or contractions are prone to both under‐ and overtriage [28, 29]. By evaluating each CCA separately, we have been able to identify potential improvement areas of GOTS. Adding a pain scoring scale might reduce the subjectiveness of pain. Adding anamnestic questions such as previous preterm birth or known placenta previa might help differentiate patients with premature contractions or bleeding into better suited triage levels. These amendments must, however, be thoroughly evaluated as the quick initial assessment must be balanced to not let triage become the entire clinical anamnesis and exam.

The process of emergency triage is more than the use of a structured support system, rather it may be considered a working method. Although triage systems aim to enable a non‐subjective and structured initial assessment, previous research shows that certain traits such as courage, confidence, and rationality of the triage nurse are imperative for adequate triage [30]. Additionally, triage performance has been shown to depend on several factors such as factual knowledge and triage experience [28, 29]. Hence, triage systems are inevitably contextual in their practical use, and patient management following the triage process must be evaluated to assess whether the information produced from the triage system is applicable and relevant in the current context. One might argue that instead of the validity of the triage system itself, it is rather the validity of the information produced by the contextual triage process that is assessed.

Previous research on validity in OTSs is scarce, lacking a standard validation approach. Research on non‐OTSs has presented a wide variety in sensitivity and specificity measures, without concluding any standard cut‐off levels. Standard acuity outcome measures must be used for an initial sorting of appropriately and inappropriately triaged patients. In our previous study, a modified Delphi method was applied to develop a set of acuity outcome measures for validation of OTSs [31]. As emphasized in this previous study, the applicability of the acuity outcome measures must be assessed for each context, and the acuity outcome measures should be used as a template for further validation and comparison.

### 4.1 Strengths and limitations

A major strength is the relatively large consecutive cohort evaluated, covering seasonal changes of infectious diseases. The study is strengthened by the applied set of standard acuity outcome measures, enabling comparative studies with other OTSs. Another strength is that the validity assessment went beyond numerical sensitivity and specificity and included a detailed assessment for different CCAs.

A major limitation is that common to all retrospective studies: the inability to control information assessment. Lacking information may be replaced, as with the 21 missing triage levels in this cohort. Nevertheless, we were dependent on information in the administrative systems and have therefore missed potentially important visual and verbal information as with cases with obvious clinical re‐triaged after CTG. Another limitation is that seven of the 15 CCA groups contained less than 50 patient assessments, impairing conclusions on sensitivity and specificity for those CCAs. For future research, one might consider studying each CCA individually to enable a richer substrate of information; however, this was beyond the scope of this study.

## 5 CONCLUSION

With an appropriate triage level in 96% of the patient visits and a sensitivity and specificity of 62% and 98%, respectively, we conclude that GOTS is a valid triage system in the studied context. Areas of improvement have also been identified. Furthermore, we suggest a two‐phase validation process with standardized acuity outcome measures and deepened chart reviews to assess validity of obstetric triage systems in a given obstetric context.

## AUTHOR CONTRIBUTIONS

Linnéa Lindroos and Verena Sengpiel planned the study. Linnéa Lindroos performed the chart reviews and Erica Ginström Ernstad performed the verification review. Linnéa Lindroos, Erica Ginström Ernstad, Staffan Nilsson, and Verena Sengpiel analyzed the results. Linnéa Lindroos wrote the first draft. Linnéa Lindroos, Erica Ginström Ernstad, Staffan Nilsson, and Verena Sengpiel have revised and approved the manuscript for publication.

## CONFLICT OF INTEREST STATEMENT

Linnéa Lindroos was the lead creator of the Gothenburg Obstetric Triage System during 2016–2017. The other authors declare no conflicts of interest.

## ETHICS STATEMENT

The study was approved by the Swedish Ethical Review Authority on October 8, 2018 (reference number 783‐18), with an amendment on November 10, 2020 (reference number 2020‐04988).

## Data Availability

Data are available upon request.
